# Pyloric Atresia in a Neonate With Epidermolysis Bullosa: A Case Report

**DOI:** 10.1002/ccr3.9685

**Published:** 2024-12-05

**Authors:** Naoya Sakamoto, Kouji Masumoto, Tomohiro Aoyama, Kazuki Shirane, Yusuke Homma

**Affiliations:** ^1^ Department of Pediatric Surgery, Institute of Medicine University of Tsukuba Ibaraki Japan; ^2^ Department of Dermatology, Institute of Medicine University of Tsukuba Ibaraki Japan

**Keywords:** epidermolysis bullosa, neonate, pyloric atresia, surgery

## Abstract

When surgery is performed in patients with EB, risks of blisters and epidermal detachment are always present. The Heineke‐Mikulicz pyloroplasty cannot always be performed because of anatomical constraints. In such cases, it is necessary to select a more time‐consuming surgical procedure (i.e., Roux‐en‐Y gastrojejunal anastomosis) with adequate fluid management.

**Abbreviations**
EB: epidermolysis bullosa
PA: pyloric atresia

## 1 Introduction

Epidermolysis bullosa (EB) is a rare disorder that affects approximately 1 in 100,000 people and can be complicated by congenital pyloric atresia (PA). When PA is associated with EB, the risk of skin blisters worsens. The Heineke–Mikulicz technique is often used to shorten operative time. Roux‐en‐Y gastrojejunal anastomosis requires a longer operative time than the Heineke–Mikulicz procedure. Therefore, this procedure is rarely used in cases of EB. However, there are cases in which the duodenum cannot be mobilized, and there are few reports on the surgical techniques applied in such cases. We herein report a neonate with PA who underwent Roux‐en‐Y gastrojejunal anastomosis. This manuscript was prepared according to CARE guidelines (https://www.care‐statement.org).

## 2 Case History

**FIGURE 1:**
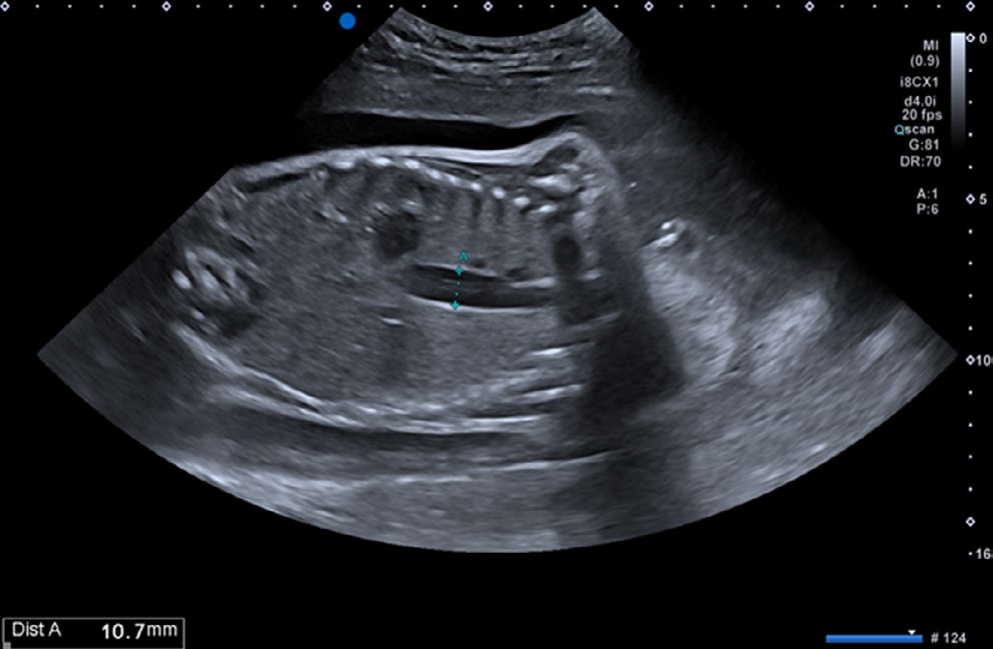
Prenatal ultrasonography showed a dilated esophagus and gastric bubble.

The patient was noted to have a dilated esophagus and gastric bubble (Figure 1) during the prenatal period, and her mother simultaneously showed excessive amniotic fluid. During the fetal period, there is a possibility of digestive tract atresia. There were no signs of snowstorms in the amniotic fluid, and it was not possible to diagnose epidermolysis bullosa during the fetal period. As a prenatal plan, we decided to wait for the fetus to grow as much as possible and then have a natural birth. She was born at 38 weeks and 0 days of gestation with a birth weight of 1740 g.

## 3 Differential Diagnosis

**FIGURE 2:**
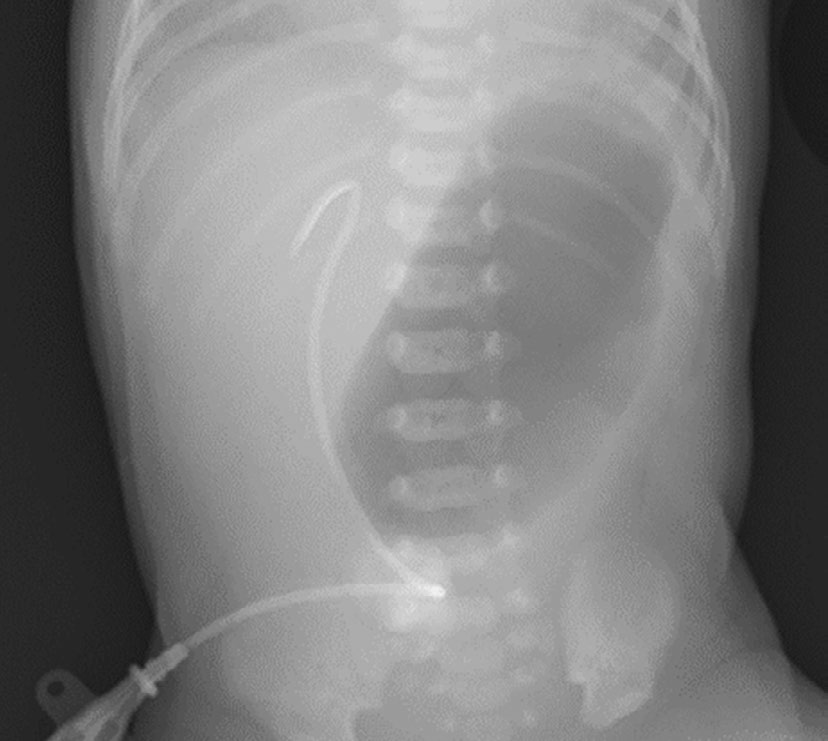
The image shows abdominal X‐ray findings of gastric dilatation and no evidence of gas in the intestinal tract beyond the duodenum.

**FIGURE 3:**
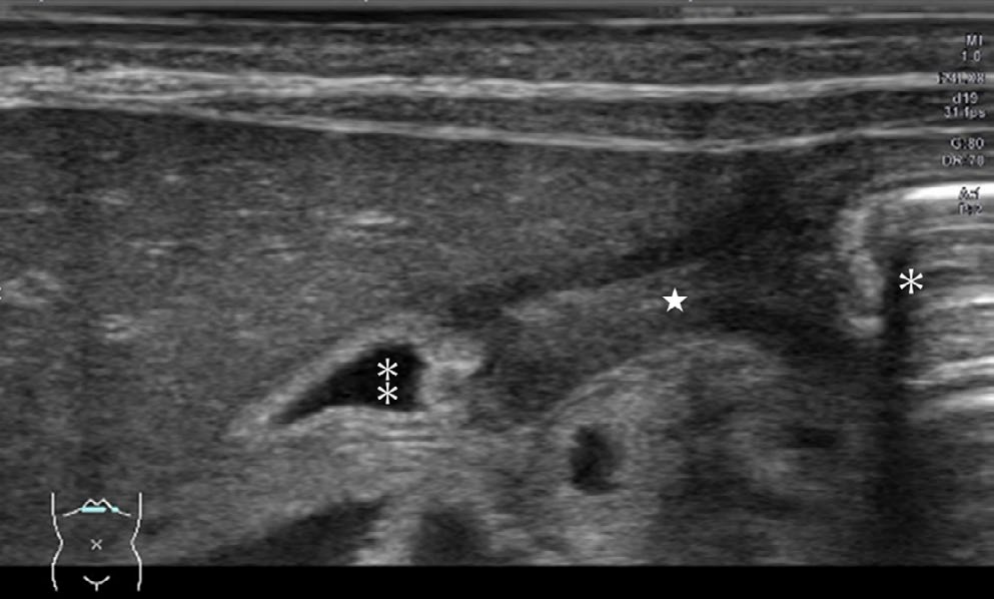
The image shows the pylorus with no passage of gastric contents (★). The right side (✽) is the gastric side, the left(⁑) is the duodenal side.

After birth, EB was suspected due to epidermolysis of the face and extremities. Abdominal radiography revealed gastric dilatation, with no evidence of gas in the intestinal tract beyond the pylorus (Figure 2). Based on these findings, pyloric stenosis or pyloric atresia were considered as possible differential diagnoses. In addition, no passage of gastric contents through the pylorus was observed on ultrasound (Figure 3), thus suggesting pyloric atresia (PA). Therefore, urgent pyloroplasty is necessary to resolve this condition.

## 4 Conclusion and Results

Before the operation, a peripherally inserted central venous catheter (PICC) was inserted via the umbilical cord. However, postoperative fluid and nutritional management was difficult with a PICC catheter alone. Therefore, it was essential to insert a Broviac catheter before pyloroplasty.

Regarding the surgical procedure, we planned to perform intubation, followed by insertion of a Broviac catheter and finally pyloroplasty. A neonatologist performed intubation. The intubation tube could not be fixed with tape; therefore, it was fixed by sewing it to the corner of the mouth with a nylon suture. A Broviac catheter was inserted using the cut‐down method. After identifying the right external jugular vein using ultrasound, the vessel was incised, and a 2.7‐Fr Broviac catheter was inserted. After creating a subcutaneous tunnel, the catheter extended from the anterior chest and then secured using a medical bond rather than thread to prevent bacterial invasion of the wound.

**FIGURE 4:**
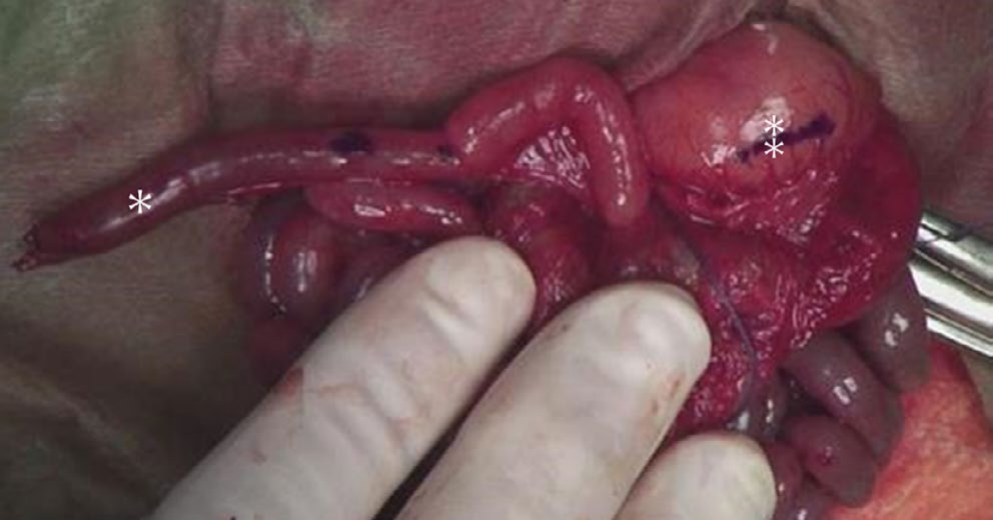
The photo shows the anastomotic site between the Roux‐limb (*) and the stomach(⁑). There was no fragility in the intestinal wall.

**FIGURE 5:**
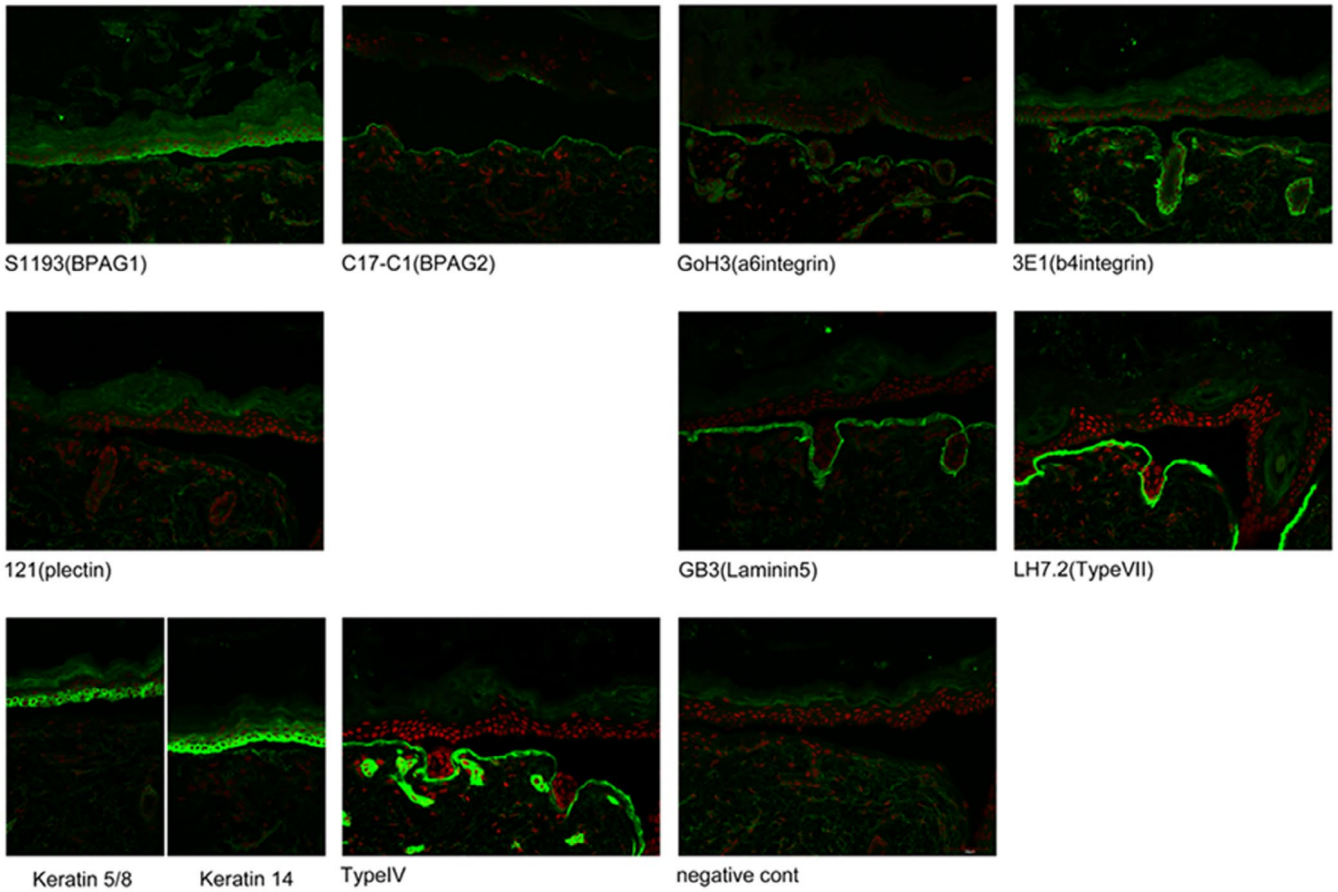
This photograph shows fluorescent immunization of the skin. Only the plectin area (left side of the center column) showed no fluorescence.

A right upper abdominal incision was made, and the skin was intact. The pylorus was located more posteriorly than usual. The duodenum must be fully mobilized to perform the Heineck–Mikulicz maneuver. However, mobilization of the duodenum was impossible deep into the abdominal cavity. This was because the child's body size was physically small, and the intestinal lumen was not sufficiently developed. This may be the result of pyloric stenosis, which prevents the passage of fluid through the intestinal lumen. Therefore, we decided to perform Roux‐en‐Y gastrojejunal anastomosis instead of Heineke‐Mikulicz pyloroplasty. In this operation, anastomosis between the stomach and jejunum was effortless while directly looking at them, and no fragility of the intestinal wall was observed (Figure 4). A 15‐cm Roux‐limb was elevated to perform the gastric jejunal anastomosis. Anastomosis was performed using 4–0 absorbable monofilament sutures with an Albert procedure. An elemental diet (ED) tube was inserted 20 cm from the anastomosis during the operation. The tip of the ED tube was placed beyond the Y‐limb anastomosis site. A skin biopsy was simultaneously performed (Figure 5), and an EB simplex with plectin deficiency was diagnosed. Postoperatively, the passage from the stomach to the small intestine is smooth. Postoperative enteral nutrition was provided via both ED and intragastric tubes during the early postoperative period. Postoperative anemia was also observed; however, iron and blood transfusions were administered via a Broviac catheter. The postoperative wound healing was good. However, on postoperative day (POD) 36, the patient developed sepsis from blisters and died on POD 64. The results of the genetic test were obtained after the patient's death. The patient's genetic mutation was a compound heterozygous mutation in PLEC. One mutation was inherited from the mother, and the other was a new *de novo* mutation. The parents were explained by a pediatric genetic specialist. The recurrence rate for the next child was extremely low, and the child's carrier rate was 50%.

## 5 Discussion

EB is a very rare disorder that affects approximately 1 in 100,000 people [1], and it can be complicated by congenital pyloric atresia (PA). EB is classified into three types: simplex with lesions in the epidermis, junctional with lesions in the basement membrane zone, and dystrophic with lesions in the dermis [2]. For a more detailed classification of EB, David et al. proposed a new system that sequentially considers the major types of EB, phenotypic characteristics, mode of inheritance, target proteins and their relative expression in the skin, genes and mutations involved, and, if possible, specific mutations and their locations [3]. Our case was a simple type of EB caused by a decreased interaction between plectin and type 17 collagen [4]. Such plectin gene abnormalities cause not only PA but also muscular dystrophy [5].

The treatment mainly targets symptoms. There is no radical cure for EB yet. Recently, two cases of stem cell therapy have been reported, but without success [6]. The first hurdle is the acute phase. To enable the patient to survive the acute phase, it is essential to administer fluids and antibiotics. Therefore, we chose to perform surgical treatment. In the present case, we chose to insert a Broviac catheter and simultaneously place an ED tube intraoperatively.

Once the acute phase is reached, skin gradually regenerates [7]. After the acute phase of EB, patients experience severe pain [8] and malnutrition [9, 10]. In patients with EB, malnutrition is caused by both a decreased nutrient intake and increased metabolic and nutrient requirements [11]. Anemia is a common complication of severe EB [12]. These guidelines clearly state that early nutritional administration is essential [13]. To improve these clinical courses, it is important to maintain both a drip infusion line and enteral nutrition route, just as in the acute phase. These clinical courses justify performing the surgery on the patient.

In the case of this disease, where the skin is fragile, surgeons should consider the impact of surgery. The key points of this case were the insertion of the Broviac catheter and pyloroplasty. Although insertion of the Broviac catheter required the creation of a subcutaneous tunnel and skin incision, our patient also required blood transfusion and iron supplementation, which we were able to provide with the help of the Broviac catheter. The skin showed good post‐operative wound closure. Therefore, placement of the Broviac catheter has more advantages than disadvantages.

We regard as the Heineke‐Mikulicz pyloroplasty as the first option to minimize the operative time, and gastroduodenal anastomosis is the secondary choice [14]. A time‐consuming procedure (i.e., Roux‐en‐Y gastrojejunal anastomosis) has been reported [15] but should be avoided. However, we should not forget that those operations cannot always be performed, as in our case. Our case suggests that appropriate intraoperative fluid management can make these time‐consuming surgical procedures feasible. Although the EB skin is fragile, the intestinal wall has a normal structure. If necessary, intestinal anastomosis should be performed.

In any surgical procedure, special attention should be paid to postoperative skin management. Despite the efforts of healthcare providers, the average life expectancy of patients with EB associated with PA is 11 months [14]. The cause is not related to the surgical technique but instead tends to be associated with inflammation, sepsis, or malnutrition [16, 17].

In our case, although wound healing was not problematic, the patient developed sepsis from blisters on the entire body. In the end, no matter which surgical procedure is chosen, advanced skin management in the postoperative period is important.

## Author Contributions

**Naoya Sakamoto:** conceptualization, writing – original draft, writing – review and editing. **Kouji Masumoto:** writing – review and editing. **Tomohiro Aoyama:** writing – original draft. **Kazuki Shirane:** writing – original draft. **Yusuke Homma:** investigation.

## Consent

Written informed consent was obtained from the parents of the patients according to the journal guidelines. Since the patient is a neonate, consent was obtained from the parents.

## Conflicts of Interest

The authors declare no conflicts of interest.

## Data Availability

Data sharing is not applicable to this article as no new data were created or analyzed in this study.
